# Wearable and Mobile Technologies for the Evaluation and Treatment of Obsessive-Compulsive Disorder: Scoping Review

**DOI:** 10.2196/45572

**Published:** 2023-07-18

**Authors:** Adam C Frank, Ruibei Li, Bradley S Peterson, Shrikanth S Narayanan

**Affiliations:** 1 Department of Psychiatry and Behavioral Sciences Keck School of Medicine University of Southern California Los Angeles, CA United States; 2 Division of Child and Adolescent Psychiatry Children's Hospital Los Angeles Los Angeles, CA United States; 3 Ming Hsieh Department of Electrical and Computer Engineering Viterbi School of Engineering University of Southern California Los Angeles, CA United States

**Keywords:** wearable, smartphone, obsessive-compulsive disorder, OCD, digital, phenotype, biomarker, mobile phone

## Abstract

**Background:**

Smartphones and wearable biosensors can continuously and passively measure aspects of behavior and physiology while also collecting data that require user input. These devices can potentially be used to monitor symptom burden; estimate diagnosis and risk for relapse; predict treatment response; and deliver digital interventions in patients with obsessive-compulsive disorder (OCD), a prevalent and disabling psychiatric condition that often follows a chronic and fluctuating course and may uniquely benefit from these technologies.

**Objective:**

Given the speed at which mobile and wearable technologies are being developed and implemented in clinical settings, a continual reappraisal of this field is needed. In this scoping review, we map the literature on the use of wearable devices and smartphone-based devices or apps in the assessment, monitoring, or treatment of OCD.

**Methods:**

In July 2022 and April 2023, we conducted an initial search and an updated search, respectively, of multiple databases, including PubMed, Embase, APA PsycINFO, and Web of Science, with no restriction on publication period, using the following search strategy: (“OCD” OR “obsessive” OR “obsessive-compulsive”) AND (“smartphone” OR “phone” OR “wearable” OR “sensing” OR “biofeedback” OR “neurofeedback” OR “neuro feedback” OR “digital” OR “phenotyping” OR “mobile” OR “heart rate variability” OR “actigraphy” OR “actimetry” OR “biosignals” OR “biomarker” OR “signals” OR “mobile health”).

**Results:**

We analyzed 2748 articles, reviewed the full text of 77 articles, and extracted data from the 25 articles included in this review. We divided our review into the following three parts: studies without digital or mobile intervention and with passive data collection, studies without digital or mobile intervention and with active or mixed data collection, and studies with a digital or mobile intervention.

**Conclusions:**

Use of mobile and wearable technologies for OCD has developed primarily in the past 15 years, with an increasing pace of related publications. Passive measures from actigraphy generally match subjective reports. Ecological momentary assessment is well tolerated for the naturalistic assessment of symptoms, may capture novel OCD symptoms, and may also document lower symptom burden than retrospective recall. Digital or mobile treatments are diverse; however, they generally provide some improvement in OCD symptom burden. Finally, ongoing work is needed for a safe and trusted uptake of technology by patients and providers.

## Introduction

### Background

The use of smartphones and wearable devices has increased recently, with an estimated 87% of adults in the United States carrying a smartphone [1] and 1 in 5 Americans using a wearable device [2]. Smartphones provide a near-constant connection to the internet and contain a suite of sensors for estimating parameters such as location, movement, and ambient sound levels [3]. Further, apps are continually being developed for smartphones that range in design from games to music and video streaming services to social media to health monitoring. Data collection from smartphones can be passive, occurring without user awareness or input (eg, accelerometry measurements), or active, where the user is engaged and directly contributes to data collection (eg, answering questionnaires or prompts). Wearable devices are a technology that directly connect to the human body and can sense aspects of physiology (eg, heart rate, oxygen saturation, glucose levels, and lactate levels) or behavior (eg, step count, time of sleep onset, and amount and type of exercise completed) and include items such as wrist-based monitors (eg, fitness trackers and smart watches), smart clothes (eg, shirts and shoes), skin patches, eyeglasses, and contact lenses (see Chan et al [4], Kim et al [5], and Zhang et al [6] for review of this technology). These devices and their associated apps are increasingly finding applications in health and medicine [3-8]. Examples include glucose monitoring in patients with diabetes [5], activity sensing in patients with heart failure [9], and lung function monitoring in patients with chronic obstructive pulmonary disease [10].

In psychiatry, use cases for wearable sensors and smartphone-based apps range in type, design, function, and objective [11,12]. Broadly, these technologies have been used to (1) detect and monitor symptoms [13-16]; (2) estimate diagnostic class, illness severity, and risk for relapse [17-20]; (3) predict response to treatment [21-23]; (4) and deliver digital interventions [24,25]. Populations in which wearable and smartphone-based technologies have been investigated and implemented vary: studies in nonclinical populations have sought to broadly promote and track mental health [26,27]. Within clinical samples, a multitude of psychiatric conditions have been explored, including depression [15,20-22], anxiety [28], schizophrenia [24], bipolar disorder [13,14,17], social anxiety [19], and obsessive-compulsive disorder (OCD) [29]. The extent of the uptake of these technologies in psychiatry varies across conditions, and we are interested in understanding the landscape of the literature covering this within the domain of OCD.

OCD is a chronic and prevalent psychiatric disorder characterized by intrusive and distressing thoughts, images, impulses, and repetitive or ritualistic behaviors [30]. OCD is considered one of the most disabling psychiatric disorders [31] and exacts a significant personal [32] and societal economic toll [33]. The course of OCD is chronic and fluctuating for many individuals [34,35], and treatment response typically hovers near 50% [36,37]. The fluctuating nature and limited treatment responsiveness of OCD present a unique opportunity for wearable and smartphone-based technologies to impact the care for and treatment of individuals with OCD. The use of technology in the treatment of anxiety and obsessive-compulsive spectrum disorders [38], specifically the use of technology in assessing and treating OCD [29], has recently been reviewed. In both reviews, the authors found heterogeneity in the implementation of technology in the care of individuals with OCD as well as an opportunity for advancing research and clinical care.

### Objectives of This Review

Given the speed at which new technologies are developed and implemented in clinical settings, a continual reappraisal of this field is needed. In this scoping review, we sought to map the literature on the use of wearable devices and smartphone-based devices or apps in the assessment or monitoring of OCD symptoms and treatment of OCD. Regarding treatment, we focused on novel interventions and excluded studies on the mobile implementation of standard psychotherapy (such as cognitive behavioral therapy [CBT] with exposure-response prevention [ERP]). We aimed to assess domains in which wearable and mobile technologies have had an impact on OCD care while also identifying areas for continued improvement and innovation within this realm.

## Methods

### Study Design

Given our objectives, the known heterogeneity in implementing technology in individuals with OCD, and the focus on emerging innovative interventions, a scoping review is the most appropriate synthesis approach. The purpose of a scoping review is to identify all available evidence to assess the breadth, depth, and nature of research activity in a topic of interest, and it is particularly useful in rapidly mapping evidence in emerging topics while maintaining rigorous search and study selection processes [39].

The protocol for this review was preregistered at the Open Science Foundation on August 9, 2022 [40]. We consulted a research librarian at the University of Southern California regarding scoping review protocols, topic development, search strategies, and data management. Keywords were initially identified from recent literature reviews relevant to the topic and preliminarily tested using the University of Southern California library database and Google Scholar (Google LLC). We included additional search terms yielded from discussion between the authors. We conducted our initial search in July 2022, with an update in April 2023; we searched multiple databases, including PubMed, Embase, APA PsycInfo, and Web of Science, with no restriction on original study design or publication period, using the following search strategy: (“OCD” OR “obsessive” OR “obsessive-compulsive”) AND (“smartphone” OR “phone” OR “wearable” OR “sensing” OR “biofeedback” OR “neurofeedback” OR “neuro feedback” OR “digital” OR “phenotyping” OR “mobile” OR “heart rate variability” OR “actigraphy” OR “actimetry” OR “biosignals” OR “biomarker” OR “signals” OR “mobile health”). Retrieved records were entered into the Covidence review software (Veritas Health Innovation), and duplicate records were removed.

### Inclusion and Exclusion Criteria

As recommended by the Joanna Briggs Institute Reviewers’ Manual for scoping reviews, we used the Population, Concept, and Context framework to inform our inclusion and exclusion criteria [41].

The inclusion criteria for study population are as follows: individuals with OCD as the primary diagnosis and of any age and sex.

Individuals who did not have OCD as a primary diagnosis were excluded from the study to avoid confounding population factors. We applied no sex or age restrictions because OCD can affect any sex and can develop at any age, including in childhood.

The inclusion criteria for study concept are as follows: biobehavioral technology or smartphone-based technology involved in the assessment or monitoring of OCD symptoms or treatment of OCD and does not use CBT and ERP.

We excluded CBT and ERP digital implementation studies because recent reviews have already covered these interventions [29,42,43].

The inclusion criteria for study context are as follows: any care setting, including inpatient, outpatient, or natural environment (eg, at home, work, or school), and English-language studies.

OCD symptoms can be potentially tracked and treated in multiple environments, including inpatient, outpatient, or naturalistic settings, depending on the severity of symptoms and the specific needs of the individual. Therefore, we chose not to restrict our search criteria to a specific setting. We included full-text English-language studies and excluded studies with only an English translation of the abstract to ensure alignment with the inclusion and exclusion criteria and appropriately extract all relevant items. In addition, our preliminary search indicated that most studies used various metrics to monitor OCD symptom burden, so not all conceptual results of interest were included in the abstract. Finally, our preliminary search yielded only a few non–English-language studies.

We decided to exclude review articles, meta-analyses, conference abstracts, and thesis defenses because these either miss elements relevant to our extraction or lack the academic rigor of the peer-review process. Given the long history of biobehavioral technology research and implementation in health care, we considered any publication date.

Together, based on the Population, Concept, and Context framework, the following study inclusion criteria were used: OCD is a primary diagnosis, studies conducted in any care setting, participants of any age and sex, study uses biobehavioral technology or smartphone-based technology, study is peer reviewed, study contains original content, and study is in the English language. Exclusion criteria were as follows: non-OCD primary diagnosis or nonclinical population; non–mobile-based technology; CBT and ERP digital implementation study; and studies that are review articles, meta-analyses, conference abstracts, or thesis defenses. No restrictions were placed on the date of publication of the included studies.

Two authors, AF and RL, screened all studies separately using the blinded screening feature of Covidence; consensus was achieved through discussion between authors for any records with conflicting screening. From this initial screening, full texts from relevant records were obtained. A total of 3 additional studies were identified for full-text review from the reference lists of other reviewed studies. Finally, AF and RL independently extracted the relevant articles and came to consensus on the final extracted items through regular discussion.

## Results

### Overview

**Figure 1:**
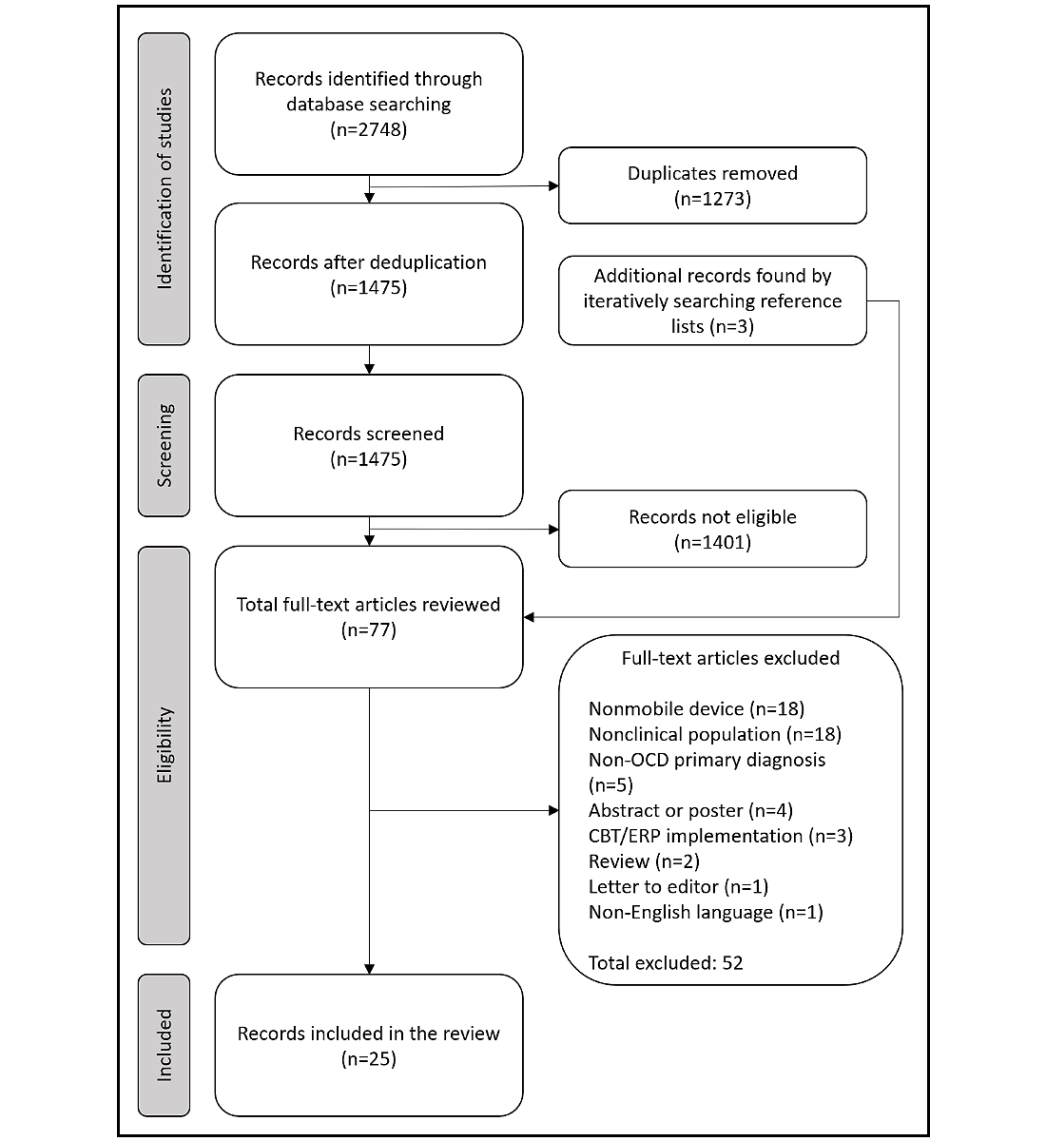
PRISMA (Preferred Reporting Items for Systematic Reviews and Meta-Analyses) diagram. CBT: cognitive behavioral therapy; ERP: exposure-response prevention; OCD: obsessive-compulsive disorder.

The scoping review was conducted using the Covidence review software, which facilitates the collation of citations with automatic deduplication, allows for blinded screening and review of articles by individual reviewers, tracks articles through the review process, and records reasons for study exclusion. Figure 1 shows the results of the systematic search, study screening, and review process conducted in Covidence. A total of 2748 records were identified across the 4 databases indicated earlier. Following the removal of duplicate records (1273/2748, 46.32%), 1475 (53.68%) of the 2748 studies remained for title and abstract screening. After screening 1475 studies, 1401 (94.98%) studies were found to be ineligible for inclusion, leaving 74 (5.02%) studies for full-text review; an additional 3 studies were identified from the reference list of the reviewed studies, resulting in a total of 77 studies undergoing full-text review. From these 77 studies, 52 (68%) were excluded, mostly studies using nonmobile devices and assessing nonclinical populations or individuals without a diagnosis of OCD. Studies were also excluded if they were conference abstracts or posters, studied the digital implementation of CBT and ERP, were not peer reviewed or were a review, or were not English-language studies.

Finally, data were extracted from a total of 25 (N=77, 32%) studies. The key findings from these studies, including the type of wearable device or smartphone app, the method of data collection (eg, active vs passive), whether an intervention was implemented, and the overall study results, are discussed in the tables.

### Studies Without Digital or Mobile Interventions and With Passive Data Collection

We broadly divided the studies into those that contained digital or mobile interventions and those that did not. We further divided studies that did not include a digital or mobile intervention based on whether they collected mobile or wearable data completely passively or had an active data collection component (ie, requiring participants to enter data or directly engage with an app). Table 1 lists studies lacking a mobile or digital intervention and collecting mobile or wearable data in a passive manner.

**Table 1:** Studies lacking digital or mobile interventions with passive mobile or wearable data collection, organized by publication year.

Study (author, year, country)	Population (age range [years])	Technology	Mobile or wearable data collected and collection method	Mobile or wearable collection method setting	Nonmobile or wearable data collected and collection method	Study aim	Main results
Millet et al [44], 1998, France	Adult men (26-43)	Actimeter (Gaewilher Electronic)	Motor activity: passive collection through actigraphy	Inpatient	Plasma melatonin: active collection Plasma cortisol: active collection Axillary temperature: active collection Symptom burden: active collection through self-report	To assess for differences in circadian variations in plasma melatonin, plasma cortisol, axillary temperature, motor activity, and obsessive-compulsive and depressive symptoms between patients with OCD^a^ and controls	Circadian variation in Hospital Anxiety Depression scale scores No significant differences in other measures between OCD and control groups
Alfano and Kim [45], 2011, United States	Youth males and females (7-11)	ActiGraphGT1M (ActiGraph)	Motor activity: passive collection through actigraphy	Natural environment	Sleep metrics: active collection through parent report Sleep metrics: active collection through self-report	To assess for differences in sleep patterns in children with OCD compared with controls using objective measures	Differences in actigraphy measures of sleep in the OCD group, including reduced total sleep time, increased wake after sleep onset, and increased duration of awakenings Negative correlation between total sleep time and CY-BOCS^b^ in the OCD group.
Drummond et al [46], 2012, England	Adult men and women (20-62)	Actiwatch-L (CamNtech Ltd)	Motor activity: passive collection through actigraphy	Inpatient	Sleep metrics: active collection through nursing report Sleep metrics: active collection through self-report	To determine the acceptability, reliability, and validity of using actigraphy to assess sleep patterns in inpatients with severe, refractory OCD	59% (36/61) of patients who were admitted agreed to participate in the study. 81% (29/36) of patients wore the actigraph for 10-20 days, 8% (3/36) wore the actigraph for up to 10 days, and 11% (4/36) were unable to wear the actigraph. Delayed sleep phase detected by actigraphy showed good agreement with nursing report and self-report.
Pittig et al [47], 2013, United States	Adult men and women (not specified)	LifeShirt system (VivoMetrics)	Electrocardiogram: passive collection Respiration: passive collection Postural data: passive collection	Outpatient	Symptom burden: active collection through self-report Task-related subjective units of distress: active collection through self-report	To assess for differences in HR^c^ and HRV^d^ in patients with anxiety disorders at rest, during stress, and during relaxation and to determine relationships with demographic or clinical variables	At baseline and during hyperventilation, lower high-frequency HRV in anxiety disorder group Greater HR during hyperventilation in PD^e^ and GAD^f^ Medication status impacted HRV in patients with OCD Age and sex related to multiple physiological variables
Bussing et al [48], 2015, United States	Youth males and females (7-17)	Actical (Mini Mitter)	Motor activity: passive collection through actigraphy	Natural environment	Sleep metrics: active collection through self-report Activation symptoms: active collection through parent report	To determine whether actigraphy can detect SSRI^g^ activation syndrome in youths with OCD relative to parent-rated measures	35% of daytime and 20% of nighttime actigraphy data were missing. Female sex associated with lower daytime activity. Parent report of daytime and nighttime activities was associated with average activity.
Donse et al [49], 2017, Netherlands	Adult men and women (not specified)	Actiwatch Spectrum Plus or Actiwatch 2 (Respironics-Philips)	Motor activity: passive collection through actigraphy	Natural environment	Sleep metrics: active collection through self-report	To assess for sleep disturbances in adults with OCD using actigraphy and self-report and to determine whether sleep disturbance can predict responsiveness to rTMS^h^ treatment	Difference in self-report measures of sleep disturbance in patients with OCD Difference in actigraphic measures of sleep disturbance in patients with OCD Circadian rhythm sleep disorder model predicted rTMS treatment nonresponse.
Jaspers-Fayer et al [50], 2018, Canada	Youth males and females (8-18)	Fitbit Flex (Fitbit Inc)	Motor activity: passive collection through actigraphy	Natural environment	Sleep metrics: active collection through parent report Sleep metrics: active collection through self-report	To assess for sleep disturbances in children and adolescents with OCD using actigraphy and parent report and self-report	72% (18/25) of patients with OCD compared with 15% (4/26) of controls met criteria for sleep disturbance by parent report. Actigraphy and self-report found longer times between going to bed and falling asleep and longer wake after sleep onset in patients with OCD.
Coles et al [51], 2020, United States	Adult men and women (not specified)	Micro Motionlogger watch (Ambulatory Monitoring Inc)	Motor activity: passive collection through actigraphy	Natural environment	Salivary melatonin: active collection Sleep metrics: active collection through self-report	To assess for differences in sleep parameters between adults with OCD and control adults using self-report, salivary melatonin levels, and wrist actigraphy and to determine whether sleep parameters correlate with symptom burden	40% (6/15) of patients with OCD met criteria for DSWPD^i^. Dim light melatonin onset occurred later in patients with OCD. Actigraphy data closely mirrored self-report sleep metrics.
Cox and Olatunji [52], 2022, United States	Adult men and women (18-53)	ActiGraph wGT3X-BT (ActiGraph)	Motor activity: passive collection through actigraphy	Natural environment	Sleep metrics: active collection through self-report OCD severity: active collection through self-report	To examine functional relationship between measures of sleep and delayed circadian rhythms in patients with OCD and its association with OCD symptom severity	Circadian rhythms are delayed in patients with OCD compared with control patients without psychiatric diagnosis. Measures of delayed circadian rhythms were associated with OCD symptoms. Measures of sleep disturbance were not significantly different between individuals with OCD and HC^j^, including objective sleep time measured from actigraphy, except for higher insomnia symptoms, which were associated with higher OCD symptoms. MEQ^k^ and DSWPD mediate OCD symptom severity through insomnia in mediation modeling.
Thierfelder et al [53], 2022, Germany	Youth males and females (13-17)	Movesense HR2, electrocardiogram chest belt (Suunto); Opal, wrist-based sensor (APDM Inc); *Look!*, custom-built eye tracker with 2 infrared and 1 field camera; Microsoft Surface Pro 7, aggregator device receiving sensor signals and pushing through recording and streaming pipeline (Surface Pro 7 i7/16GB/256GB, Microsoft Corp); and Aggregator Software, custom developed software for data processing and user interface to connect, control, and record the sensors	HR: passive collection through electrocardiogram chest belt Motor activity: passive collection through wrist-based sensors Eye-tracking: passive collection through head-mounted device Data aggregation and processing: passive collection through Surface Pro and custom-built software	Outpatient	N/A^l^	Pilot study to demonstrate that the collected sensor data capture features of stress reactions, compulsive behavior, and relief from anxiety in an outpatient setting in adolescents with OCD	RMSSD^m^ (measure of HRV) decreased for all participants with increasing OCD-related stress. HR (BPM^n^) increased or remained stable with increasing stress. Relief from stress is generally accompanied by an increase in the RMSSD of HRV and decreased or stable HR. Increases in physical activity are generally accompanied by a drop in RMSSD and more elevated HR than in a stressful event. Increase in movement energy can be observed from wrist-based sensors with increasing OCD-related stress. Repetitive compulsive behavior (checking bag) captured by hand sensors showed a unique frequency distribution compared with other repetitive but noncompulsive behavior. While refraining from compulsion to wash hands, participants exposed to contamination in public bathroom fixated on the public toilet (59%), floor (27%), and sink (14%).
Gajadien et al [54], 2023, Netherlands	Adult men and women (not specified)	ActTrust (Condor Instruments)	Motor activity: passive collection through actigraphy	Natural environment	Sleep metrics: active collection through self-report	To investigate potential differences in sleep parameters between responders and nonresponders to rTMS and to examine the ability of sleep parameters to predict rTMS response	Reduction in OCD and depressive symptoms after rTMS treatment No baseline characteristics significantly different between rTMS responders and nonresponders Actigraphy parameters did not meet the effect size requirement of Cohen d ≥0.5 for inclusion as a predictor Discriminant model including subjective sleep quality, sleep latency, daytime dysfunction, and HSDQ^o^ insomnia could predict response to rTMS with an AUC^p^ of 0.813, a sensitivity of 76%, and a specificity of 50% Circadian rhythm sleep disorder model from the study by Donse et al [49] was not significant and could not be replicated in this study

^a^OCD: obsessive-compulsive disorder.

^b^CY-BOCS: Children’s Yale-Brown Obsessive Compulsive Scale.

^c^HR: heart rate.

^d^HRV: heart rate variability.

^e^PD: panic disorder.

^f^GAD: generalized anxiety disorder.

^g^SSRI: selective serotonin reuptake inhibitor.

^h^rTMS: repetitive transcranial magnetic stimulation.

^i^DSWPD: delayed sleep-wake phase disorder.

^j^HC: healthy control.

^k^MEQ: Morningness-Eveningness Questionnaire.

^l^N/A: not applicable.

^m^RMSSD: root mean square of successive differences.

^n^BPM: beats per minute.

^o^HSDQ: Holland sleep disorder questionnaire.

^p^AUC: area under the curve.

Overall, the studies in this category recruited a mix of youth and adult participants and a mix of male and female participants. A total of 2 (18%) of the 11 studies in this group collected wearable data using advanced body sensors that required monitoring in an outpatient setting. One study used a shirt embedded with sensors to collect physiological data, including heart rate and heart rate variability (HRV; calculated from a continuous electrocardiogram), respiration rate, and postural data in patients with anxiety disorders, including OCD. Briefly, the authors found reduced high-frequency HRV at baseline and during hyperventilation in patients with anxiety compared with control participants. They also found a higher heart rate in patients with panic disorder and generalized anxiety disorder during hyperventilation [47]. The other study piloted the ability of a variety of sensors to capture OCD symptomology for a larger study. The team used multiple sensors, including an electrocardiogram chest belt, wrist-based sensors, and a custom-built eye-tracking device to measure gaze fixation during OCD-induced stress as well as heart rate, HRV, and hand motor activity during planned OCD-triggering events [53]. HRV decreased with higher stress levels and increased during rest, whereas heart rate either increased or remained stable with higher stress levels and decreased during rest. Additionally, HRV and motor activity data yielded data patterns that distinguished OCD-induced stress from physical activity.

All other studies in this group used actigraphy as an objective measure of activity. Most studies (8/11, 73%) used actigraphy at night as a measure of sleep while also collecting self-report, parent report, or nursing report as additional metrics of sleep quality and quantity. A majority of studies (7/11, 64) collected actigraphy measures in a naturalistic home environment; however, 2 (18%) of the 11 studies were conducted in an inpatient setting, with 1 study focusing on individuals with treatment-refractory OCD [46] and the other study requiring inpatient hospitalization to draw concurrent blood samples [44]. In general, studies found differences in objective and subjective measures of sleep in patients with OCD, such as decreased total sleep time [45,49], increased number of awakenings after sleep onset [45,50], increased duration of awakenings [45], increased time to fall asleep [49,50], later midsleep timing [52], and presence of delayed sleep phase disorder [46,51,52]. However, 1 study provided contrary evidence on sleep disturbance, which was largely nonsignificant between participants with OCD and control participants in subjective and actigraphy measures [52]. Another study analyzed actigraphy and self-reported sleep measures between responders and nonresponders to repetitive transcranial magnetic stimulation (rTMS); the authors found that a circadian rhythm sleep disorder model could discriminate between responders and nonresponders to rTMS treatment with a sensitivity of approximately 84%. An insomnia model could not discriminate between these groups [49]. A more recent study from the same group, however, did not identify the circadian rhythm sleep disorder model as a potential predictor of rTMS response but did find measures of sleep disturbance, as measured by self-report but not actigraphy measures, to be predictive of rTMS response, with an area under the curve of 0.813, sensitivity of 76%, and specificity of 50% [54]. Other investigators have used actigraphy to measure activity in patients with OCD during daytime hours. One study reported no abnormalities in circadian variability compared with controls [44]. Another study that assessed behavioral activation from selective serotonin reuptake inhibitor treatment reported lower daytime activity in girls and an association of parent reports with actigraphy measures of activity [48].

### Studies Without Digital or Mobile Interventions and With Active or Mixed Data Collection

All the study participants in this category were adults. Ecological momentary assessments (EMAs) occur in a naturalistic setting and were used in all studies that collected mobile or wearable data in an active or mixed fashion (active and passive concurrently, albeit only Brown et al [55] used a mixed collection method) (Table 2).

**Table 2:** Studies without digital or mobile interventions and with active or mixed mobile or wearable data collection, organized by publication year.

Study (author, year, country)	Population (age range [years])	Technology	Mobile or wearable data collected and collection method	Mobile or wearable collection method setting	Nonmobile or wearable data collected and collection method	Study aim	Main results
Gloster et al [56], 2008, United States	Adult men and women (20-62)	Palm Zire 21 personal data assistant (Palm Inc)	Symptom burden: active collection through self-report Context and social interaction: active collection through self-report	Natural environment	N/A^a^	To determine the accuracy of retrospective estimates of daily OCD^b^ symptom burden and symptom covariation relative to prospectively collected EMAs^c^ in patients with OCD	Retrospective recall of OCD symptoms was generally consistent with EMA data, although there was occasional underestimation of the frequency of OCD behaviors Consistent overestimation of the covariation of symptoms with nonsymptomatic variables
Tilley and Rees [57], 2014, Australia	Adult men and women (28-54)	SMS text message–based prompts; Olympus WS-110 digital voice recorder (Olympus, Tokyo, Japan)	Symptom burden: active collection through self-report	Natural environment	N/A	To determine whether the use of EMA can provide additional diagnostic information in those with OCD	Fewer symptoms were endorsed by EMA, although new types of symptoms were reported.
Rupp et al [58], 2019, Germany	Adult men and women (not specified)	movisenseXS (movisens GmbH) implemented on Motorola Moto G2 (Lenovo)	Symptom burden: active collection through self-report Emotions related to OCD: active collection through self-report Behaviors related to OCD: active collection through self-report	Natural environment	Feasibility of EMA: active collection through self-report	To determine the feasibility and effectiveness of using EMA to assess OCD symptoms before and after psychotherapy treatment	28.11% (851/3027) of EMA responses removed during data cleansing Questions regarding acceptability, practicability, representativeness, and reactivity were rated fairly, and responses did not change before and after treatment Reductions in avoidance and obsessions following treatment
Brown et al [55], 2020, United States	Adult men and women (not specified)	Fitbit Alta (Fitbit Inc), Twilio technology (Twilio Inc), and Way to Health Platform [59]	Behavior and physiology: passive collection through Fitbit Symptom burden: active collection through self-report Social interaction and context: active collection through self-report	Natural environment	Acceptability: active collection through qualitative interview	To assess patients’ and clinicians’ perspectives on the use of a wearable biosensor and EMAs in measurement of OCD symptoms	High EMA response rate (90.2%) with moderate adherence to physical activity (57.7%) and sleep (52.2%) data collection Multiple patient qualitative themes from Fitbit and EMA use were generally positive, although there were some concerns about technology use and data accuracy Clinician themes included concerns about amount of data and integration into clinical care
Rupp et al [60], 2020, Germany	Adult men and women (not specified)	movisenseXS (movisens GmbH) implemented on Motorola Moto G2 (Lenovo)	Emotions related to OCD: active collection through self-report Behaviors related to OCD: active collection through self-report	Natural environment	N/A	To use pretreatment and posttreatment EMAs to compare the effects of 2 weeks of CR^d^ treatment on OCD with those of DM^e^ treatment on OCD	Some baseline use of therapy techniques by participants before treatment Increase in the use of psychotherapy strategies and behaviors after treatment No difference between different therapy modalities in the frequency of use, perceived difficulty, and the experience of relief after treatment

^a^N/A: not applicable.

^b^OCD: obsessive-compulsive disorder.

^c^EMA: ecological momentary assessment.

^d^CR: cognitive restructuring.

^e^DM: detached mindfulness.

A series of studies assessed whether the use of EMA could outperform retrospective symptom recall [56], uncover new OCD symptoms [57], and feasibly monitor symptoms [55]. EMA reported a slightly lower frequency [56] and burden [57] of OCD symptoms than clinician-administered Yale-Brown Obsessive Compulsive Scale (Y-BOCS) or Obsessive Compulsive Inventory-Revised, although EMA captured novel, previously unreported OCD symptoms [57]. Although patients’ perspectives on the use of EMA were generally positive, clinicians expressed some concern about the amount of data collected and the integration of EMA into clinical care [55]. Rupp et al [58] assessed the feasibility of using EMA to assess OCD symptom burden before and after participants completed detached mindfulness or cognitive restructuring psychotherapy. Participants generally rated EMA highly in terms of acceptability, practicability, and representativeness. The data were quite noisy; however, approximately 28.11% (851/3027) of the noise was removed during data cleansing [58]. A separate study assessed the results of these psychotherapy interventions and found no significant differences between the detached mindfulness and cognitive restructuring therapies in the frequency of their use, perceived difficulty, or the experience of relief after treatment [60].

### Studies With Digital or Mobile Interventions

All studies in this section include some form of mobile or digital intervention; however, we excluded studies that focused on CBT and ERP implementation. All but 1 study (8/9, 89%) also involved the collection of active or passive digital or mobile data (Table 3).

**Table 3:** Studies with digital or mobile interventions, organized by publication year.

Study (author, year, country)	Population (age [years])	Technology	Mobile or wearable data collected and collection method	Nonmobile or wearable data collected and collection method	Digital or mobile intervention	Digital or mobile intervention setting	Study aim	Main results
Le Boeuf [61], 1974, England	Adult man (49)	Portable shock box carried in jacket pocket and connected via electrodes to the forearm and base of the index finger; shock delivered if circuit completed through the immersion of hands in water	Presence of water: passive collection through shock device	Daily handwashing frequency: active collection through self-report	Shock device was turned on for specified periods to provide positive punishment for handwashing	Natural environment	To determine the efficacy of an automated shocking device in the treatment of compulsive handwashing	Decrease in daily handwashing following 2 weeks of shock box use
Olbrich et al [62], 2016, Germany	Adult man (31)	Geo-Feedback App (developed by S Olbrich)	Position: passive collection through GPS	Time to reach treatment clinic: active collection through self-report	Mobile app provides the user a notification if they have not moved a predefined distance in a given length of time	Natural environment	To determine whether smartphone-based feedback can be used to treat OCD^a^	Use of mobile app decreased the time needed to reach treatment clinic (1 mile distance) from 2 hours to 1 hour. With the addition of consistent ERP^b^ and app use, time to reach the clinic decreased to 20 minutes. Patient reported the fear of attracting attention from app notifications as a negative reinforcer.
Kashyap et al [63], 2019, India	Adult man (29)	CogTrain App (developed by P Reddy and S Mandadi)	N/A^c^	Cognitive and symptom assessments: active collection through clinician-administered	Mobile app for cognitive training (coupled with in-person therapist-guided sessions)	Natural environment	To report on the use of cognitive training as an intervention for OCD using a custom smartphone app, therapist training, and various freely available smartphone apps	Patient completed therapist-guided cognitive training, mindfulness practices, and ADL^d^ training. Patient completed cognitive training tasks and used the CogTrain App. Over 12 weeks, patient had improvement in symptom burden and improvement in some cognitive measures.
Arevian et al [64], 2020, United States	Adult men and women (18-69)	Chorus platform (Chorus Innovations Inc) and SMS text messages	Symptom burden and response to treatment: active collection through self-report	Feasibility and acceptability: active collection through clinician-administered survey	SMS text messages sent to participants to encourage adherence to treatment, remind them to take medication and engage in exposures, and provide information in addition to IOP^e^ treatment	Natural environment	To evaluate the usability of a mobile texting app, to evaluate the feasibility of app development with patients and providers, and to describe the types of texting apps developed	1787 messages sent and 80 responses received Various types of messages were created, and overall themes for messages were personalization to individuals and use of humor Most patients expressed positive feedback about the development and use of messages Themes from workgroups included treatment engagement, personalization of treatment, motivation, and after-hours care
Olsen et al [65], 2020, United States	Adult man (20s)	Activa PC+S (Medtronic) and smartphone-based EMA	Intracranial LFP^f^: passive collection through DBS^g^ system Motivation and functionality: active collection through EMA	MSIT^h^: active collection Symptom burden: active collection through clinician-administered measures	Open-loop, dual-site DBS to the bilateral VC/VS^i^ and SMA^j^	Natural environment and outpatient	To test the feasibility of combining VC/VS DBS with frequency-mismatched stimulation of the SMA in treating refractory OCD	Small decrease in Y-BOCS^k^ with cortical stimulation and small increase in MADRS^l^ PGI-I^m^ improved with the addition of cortical stimulation MSIT reaction time improved with dual-site stimulation Cortical-striatal synchrony increased with dual-site stimulation Various changes in power spectra through study Random forest model predicting PGI-I performed with 92% accuracy, and cortical-striatal gamma and theta synchrony were important features
Provenza et al [66], 2021, United States	Adult men and women (31-40)	Summit RC+S (Medtronic); Apple Watch (Apple Inc); StriveStudy mobile app (Rune Labs); Honeycomb task app; actiCAP electroencephalogram cap (Brain Products GmbH); GoPro Hero 6 (GoPro Inc); H4n Pro 4-track Portable Recorder (Zoom Corp); and AFAR^n^ computer-vision (Carnegie Mellon University)	Intracranial electrophysiology: passive collection through DBS device Heart rate, blood volume pulse, and acceleration: passive collection through Apple Watch OCD symptom severity: active collection through self-report via StriveStudy app Performance on cognitive and behavioral tasks: active collection through Honeycomb app Extracranial electrophysiology: passive collection through actiCAP Facial movements: passive collection through GoPro and AFAR Speech: passive collection through H4n recorder	Symptom burden: active collection through clinician-administered measures	Open-loop DBS to bilateral VC/VS or BNST^o^	Natural environment and outpatient	To identify the neural biomarkers of OCD through (1) the measurement of intracranial and extracranial electrophysiology, (2) self-reported OCD symptom burden, (3) objectively measured affective state, and (4) the evaluation of physiology for the purpose of developing an adaptive DBS for OCD	AFAR software estimated positive affect and head velocity, and these data were synchronized with blood volume pulse, electrocardiogram, electroencephalogram, LFP from DBS, and the acceleration of INS^p^ Increase in positive affect and subjective positive feelings during DBS programming session in participant 5 Self-reports were synchronized to at-home, wirelessly streamed DBS LFP, routine at-home tasks, and psychophysiological tasks In total, across the 3 participants, over 1000 hours of at-home intracranial physiology was recorded 1 participant completed an at-home LFP recording for 3 continuous days; 41 OCD symptom intensity ratings were collected during this period (range 0-8); LFP frequency band power was examined in the minute before and after self-report; there was a strong negative correlation between power in the delta band and OCD symptom severity in both the left (R=−0.593) and right (R=−0.557) VC/VS; correlations to planned ERP exposures in this participant were also seen in the delta band
Hawley et al [67], 2021, Canada	Adults of unspecified sex (not specified)	Muse, a consumer-grade electroencephalogram headset device with a mobile app (InteraXon Inc)	Electroencephalogram: passive collection through Muse device	Symptom burden: active collection through self-report	Muse technology–guided neurofeedback with daily guided mindfulness	Natural environment and outpatient	To determine whether technology-supported mindfulness can improve OCD symptom burden, increase self-reported mindfulness, and increase electroencephalogram-derived indicators of mind wandering	Decrease in Y-BOCS-SR^q^ in the active treatment group Increased alpha and beta electroencephalogram power in the treatment group Alpha and beta power predicted Y-BOCS-SR decrease Measures of mind wandering predicted Y-BOCS-SR
Hawley et al [68], 2021, Canada	Adults of unspecified sex (not specified)	Muse, a consumer-grade electroencephalogram headset device with a mobile app (InteraXon Inc)	Electroencephalogram: passive collection through Muse device	Symptom burden: active collection through self-report	Muse technology–guided neurofeedback with daily guided mindfulness	Natural environment and outpatient	To determine whether technology-supported mindfulness training is associated with decreased cognitive vulnerability, improved attention, reduced OCD symptom burden, and the existence of a relationship between electroencephalogram-derived markers of attention and clinical variables	Decrease in Y-BOCS-SR in the active treatment group Increased alpha and beta electroencephalogram power in the treatment group OBQ^r^ perfectionism or certainty and importance or control and Y-BOCS-SR bidirectionally predicted changes in each value Alpha power and OBQ perfectionism or certainty bidirectionally predictive of each other
Fridgeirsson et al [69], 2023, Netherlands	Adult men and women (30-69)	Medtronic Percept or Activa PC+S with 3389 DBS leads (Medtronic)	Intracranial LFP: passive collection through DBS system	Symptom burden: active collection through self-report	Open-loop DBS to bilateral vALIC^s^, although DBS was not active during the study period	Outpatient	To identify an electrophysiologic biomarker of OCD symptoms in adults implanted with DBS in the vALIC through machine learning approaches	Obsession induction increased VAS^t^ scores for anxiety, agitation, obsession, and compulsions Power in all examined frequency bands increased during compulsions and relief state relative to baseline Total balanced accuracy of predicting individuals from baseline LFP data was 18.9% for a boosted trees model and 32.6% for a deep learning model compared with a chance level of 9% (*P*<.05) Patient-specific models showed an average accuracy of 32.5% in predicting the symptom state of individual patients using boosted trees and 38.8% accuracy using deep learning Deep learning reached an average AUC^u^ of 78.2% for compulsions, 62.1% for obsessions, 58.7% for baseline, and 59.7% for relief

^a^OCD: obsessive-compulsive disorder.

^b^ERP: exposure-response prevention therapy.

^c^N/A: not applicable.

^d^ADL: activities of daily living.

^e^IOP: intensive outpatient program.

^f^LFP: local field potential.

^g^DBS: deep brain stimulation.

^h^MSIT: multisource interference task.

^i^VC/VS: ventral capsule/ventral striatum.

^j^SMA: supplementary motor area.

^k^Y-BOCS: Yale-Brown Obsessive Compulsive Scale.

^l^MADRS: Montgomery-Asberg Depression Rating Scale.

^m^PGI-I: patient global impression of improvement.

^n^AFAR: automatic facial affect recognition.

^o^BNST: bed nucleus of the stria terminalis.

^p^INS: implanted neural stimulator.

^q^Y-BOCS-SR: Yale-Brown Obsessive Compulsive Scaleself-report.

^r^OBQ: Obsessive Beliefs Questionnaire.

^s^vALIC: ventral anterior limb of internal capsules.

^t^VAS: visual analog scale.

^u^AUC: area under the curve.

All studies applied interventions in a natural environment, with some providing constant treatment via deep brain stimulation (DBS) [65,66] and others using a 2-pronged at-home and in-clinic interventional approach [67,68]. A total of 2 (22%) of the 9 studies developed novel interventions aimed specifically at OCD symptoms. In a single-participant case report, Le Boeuf [61] created a wearable device that provided a mild electric shock to the user if an electrical circuit was completed when the user’s hands were in contact with water, presumably during a washing compulsion. The participant had severely impairing, compulsive handwashing before treatment and had a marked and durable improvement in symptom burden soon after beginning the use of the wearable device [61]. Even more notable is that this study was completed in 1974—well before the advent of smartphones or modern wearable biosensors. In another single-participant case report, Olbrich et al [62] developed a smartphone app to address severe harm-based obsessions and checking compulsions that prevented the participant from attending psychotherapy appointments. The smartphone app tracked the user’s location and sent a reminder signal if the participant had not moved a predefined distance. Use of the app reduced the time required for the participant to reach the clinic by 50% (2 hours to 1 hour for a travel distance of 1 mile); once the patient was able to re-engage in ERP (and continue to use the app), he reached the clinic in 20 minutes. He endorsed that the app served as a negative reinforcer in that he feared drawing attention to himself if the app made a signal noise [62].

A total of 2 (22%) of the 9 studies used DBS as a treatment modality, collected longitudinal intracranial physiological measures from the DBS electrodes, and collected passive and active digital and wearable metrics. In a case report of a patient receiving dual-site stimulation in the ventral capsule/ventral striatum and supplementary motor area, Olsen et al [65] found a small improvement in Y-BOCS score and more robust improvement in patient global impression of improvement following dual-site stimulation compared with single ventral capsule/ventral striatum stimulation. Unexpectedly, cortical-striatal synchrony increased with dual-site stimulation, and random forest modeling showed that cortical-striatal gamma and theta synchrony predicted patient global impression of improvement with 92% accuracy [65]. Provenza et al [66] took a multimodal approach to studying OCD by chronically recording intracranial local field potentials (LFPs) while also densely collecting other measures, including heart rate, self-report symptom burden, facial features, and speech samples. The authors presented data on the first few participants in a planned, larger study. They found significant correlations of facial affect with subjective improvement during DBS programming, negative correlations of delta band power with OCD symptom severity, and correlations of delta band power with ERP [66]. Fridgeirsson et al [69] used machine learning models to analyze LFP data collected during rest and symptom provocation in an outpatient setting while DBS was turned off. Machine learning modeling of resting LFP identified individual patients significantly above the chance level. Modeling of LFP collected during symptom provocation and relief of symptoms predicted symptom state with an average accuracy of 32.5% and 38.8% for the boosted trees and deep learning model, respectively, with the latter reaching an average area under the curve of 78.2% for compulsions, 62.1% for obsessions, 58.7% for baseline, and 59.7% for relief [69].

A total of 2 (22%) of the 9 studies from 1 research group used a mobile electroencephalogram device for monitoring during biofeedback treatment. They found that active treatment reduced Y-BOCS self-report scores and increased electroencephalogram alpha and beta power in proportion to the improvement in OCD symptoms [67]. They also found that alpha power and the perfectionism or certainty subscores from the Obsessive Beliefs Questionnaire were reciprocally associated with one another across time [68].

Arevian et al [64] developed and tested the usability and feasibility of a mobile texting app cocreated by therapists and patients for use in an OCD treatment clinic. The app prompted patients with SMS text messages, and some SMS text messages also requested patient engagement and response. The authors found that the types of SMS text messages created generally focused on personalizing treatment for the individual and using humor to aid in treatment. Approximately 80 to 90% of the patients expressed positive sentiments about the development and use of the app. Themes from working groups of therapists regarding app development and use included treatment engagement, personalization of treatment, motivation, and provision of after-hours care [64].

Finally, 1 (11%) of the 9 studies used a custom mobile phone app aimed at improving cognition in individuals with OCD; this was coupled with therapist-led treatment in a single individual with subjective cognitive complaints [63]. The authors reported that this multimodal approach was associated with improvements in subjective and objective measures of cognition.

## Discussion

### Overview

Wearable sensors and smartphone-based apps are increasingly being used in medicine and health broadly and in psychiatry specifically to monitor symptoms, diagnose diseases, and predict responses to treatment [3-5,7,8,11,12]. Given that OCD is a chronic, fluctuating condition with significant personal and economic costs, there is a unique opportunity to implement this evolving digital data collection framework to improve our understanding of disease mechanism and improve treatment and clinical outcomes [32-35]. This scoping review maps the extant literature on the use of wearable and smartphone-based technologies in tracking, diagnosing, and predicting clinical outcomes in individuals with OCD. The included studies were broadly divided into studies with digital or mobile interventions and those without. Studies without such interventions were further categorized based on whether they solely collected mobile or wearable data passively or involved an active component in data collection. The results of recent reviews of technology use in OCD do not meaningfully overlap with our results: Cooper et al [38] explored the use of technology in facilitating therapist-delivered psychotherapy in person or by webcam, assessment and prediction of OCD symptoms, and interventions in treating OCD, with the results of none of the reviewed studies overlapping ours; Ferreri et al [29] focused broadly on the use of technology in the assessment and prediction of and interventions for OCD, with the results of only 2 reviewed studies overlapping with ours [57,62].

### Principal Findings

We found several broad themes through this study. First, except for 2 (8%) of the 25 studies [44,61], the reviewed studies indicate that the use of wearable sensors or mobile apps in evaluating and treating OCD has developed within the past 15 years, with over half (15/25, 60%) of the studies having been conducted in the last 5 years. This speaks not only to the novelty of these methods in psychiatry but also to the increasing pace of adoption of mobile and wearable technologies in health and medicine. Second, regarding the types of technology, most studies using fully passive mobile or wearable data collection used actigraphy to assess sleep or, less frequently, daytime movement patterns. These studies generally reported good agreement between objective actigraphy data and patient-, parent-, or nurse-reported subjective metrics. However, no study used actigraphy as the sole measure of sleep, which may be indicative of the current limitations of actigraphy. Several studies used extensive, nonconsumer sensors and modalities, including custom-built hardware and software, to passively track OCD symptoms. These efforts highlight the potential therapeutic benefit of tracking OCD symptoms passively and the desire for higher-performance sensors that are not available in off-the-shelf solutions. Many studies that actively collect mobile or wearable data use EMA to assess OCD symptom severity and burden in a naturalistic manner. In general, EMA is well tolerated by participants and appears to uncover new OCD symptoms not reported on retrospective questionnaires, although it may underestimate the overall OCD symptom burden.

We found that mobile or digital interventions are varied and diverse. They include apps and devices that provide negative reinforcement, apps that provide cognitive training, apps facilitating bidirectional texting and SMS text messaging between providers and patients, electroencephalogram-based biofeedback devices, and open-loop DBS with concurrently recorded intracranial LFP. Studies that leveraged mobile or digital interventions were often case reports with a single male participant. The dearth of studies involving a larger and more diverse participant pool highlights the novelty of such interventions. Nevertheless, given the rapidity of technology development and adoption, we anticipate the depth and breadth of mobile and digital interventions to continue to expand with increasing speed. Finally, approaches to data privacy and security are often underreported. This is a critical issue to address given the user concerns about these technologies [70-72], the ongoing integration of technology into health care, and the potential for malicious use of data [73].

The findings of this review highlight several important considerations for future studies and the implementation of digital health technologies in clinical practice. First, the consistency and standardization of data collection and analysis are important and likely to improve both study quality and public perception of digital or wearable technology research. To facilitate this effort, future studies will benefit from the use of a conceptual framework that allows one to identify important metrics to assess, determine on what timescale to collect these measures, and decide how to implement appropriate and statistically sound analytic methods. Two commonly used and conceptually overlapping frameworks—behavioral signal processing [74] and digital phenotyping [75,76]—share important features and aims: acquisition of multimodal and ecologically valid data, selection of analytic methods suited to the acquired data, and development of models to predict clinical course and treatment response. Both approaches have been usefully implemented in psychiatric conditions as diverse as schizophrenia [77], depression [78,79], anxiety [80,81], and autism spectrum disorder [82,83].

Second, wearable- and smartphone-based studies have the potential to improve treatment outcomes through the development of intervention decision models, which are collections of strategies and policies for the evaluation and treatment of patients and are commonly used in diverse fields of medicine [84]. Decision models operate most effectively when the illness phenomenology (ie, signs and symptoms) maps onto an understanding of the pathophysiology. Wearable devices and smartphones will allow for the ongoing collection of diverse, dense, longitudinal data sets that can improve our understanding of the signs and symptoms of psychiatric disorders; in conjunction with research on the pathophysiology of mental illness, these complementary approaches will lead to the development of much-needed decision models in psychiatry [84].

Third, the included studies were conducted across the globe in countries, including India, Australia, Canada, the United States, and several European nations. Globally, smartphone use ranges from 70% to 85% of the population and is steadily increasing [85,86]. Furthermore, mental illness is prevalent throughout the world [87,88], and even within the United States, there are disparities in access to care based on race and ethnicity [89,90]. We also found that studies were conducted across age groups, from children and adolescents to those in their 60s, and in both males and females (although not all studies reported age). Taking these themes together, wearable- and smartphone-based studies can, and should, be conducted in diverse settings and populations around the world. This naturally lends itself to large, concurrent studies that are scaled up to include many more participants so that variability in measures can effectively be captured and analyzed.

Fourth, the declining costs of technology, ubiquitous use of smartphones and their associated functionalities (eg, user interface, cloud connection, and data storage and sharing), and integration of artificial intelligence for high-dimensional data processing have enabled real-time monitoring of various health-related biomarkers via wearable biosensors [6]. For instance, a recently proposed study uses an armband biosensor to passively monitor diverse physiological parameters in patients with COVID-19. An associated smartphone app receives and stores real-time data from the sensor and subsequently uploads them to a cloud-based server, where further processing occurs via machine learning. The results can then be displayed to a clinician via a web-based dashboard with an overall goal of early detection of disease progression [91]. From our review, Provenza et al [66] took a similar approach in capturing diverse streams of wearable and mobile data concurrently. They demonstrated an approach to combining these data into a broader scientific and clinical picture [66]. We anticipate that future studies in psychiatry will further integrate actively and passively collected wearable and mobile data, on-device and cloud-based storage, and real-time data extraction and analysis to produce actionable information that patients and clinicians can use to guide care.

Finally, the ethical and legal frameworks surrounding mobile and wearable data collection and use continue to evolve, particularly as the definitions of devices and apps change [92]. Currently, most devices are not regulated by the Food and Drug Administration, although their features or marketing suggest medical diagnostic capabilities; this leaves manufacturers and, potentially, physicians open to state and federal liabilities should these devices malfunction or fail to perform as advertised [93]. Simon et al [93] suggested changes to state and federal regulations to mitigate this liability, although they also note that best practices developed by physician organizations that specifically address mobile or wearable devices may reduce some legal risk.

### Strengths and Limitations

We present an overview of the use of mobile and wearable technologies in the monitoring and treatment of OCD. Our systematic approach to the literature ensured that all indexed studies were included, supported by our identification of an older study not previously captured in reviews [61]. It is possible that relevant non–English-language studies were overlooked, as we focused our review on manuscripts published in English. We divided the included studies into studies passively collecting data, studies actively collecting data, and studies implementing treatment; this decision was based on the structure and findings of these studies and was intended to highlight the current landscape of the field, although other organizational approaches could be validly implemented. We chose not to focus on digital or mobile implementation of CBT and ERP, given the existing recent reviews highlighting the literature covering CBT and ERP [29,42,43], and to identify studies reporting novel digital or mobile treatment approaches. Finally, we briefly suggest areas for ongoing consideration when designing studies and considering the clinical implementation of mobile and wearable technologies in OCD. This field is evolving rapidly, and continued publication of high-quality research is paramount for a safe and trusted uptake of technology by patients and providers.

## Abbreviations

CBT: cognitive behavioral therapy
DBS: deep brain stimulation
EMA: ecological momentary assessment
ERP: exposure-response prevention
HRV: heart rate variability
LFP: local field potential
OCD: obsessive-compulsive disorder
rTMS: repetitive transcranial magnetic stimulation
Y-BOCS: Yale-Brown Obsessive Compulsive Scale

## References

[1] Faverio M (2022). Share of those 65 and older who are tech users has grown in the past decade. Pew Research Center.

[2] Vogels EA (2020). About one-in-five Americans use a smart watch or fitness tracker. Pew Research Center.

[3] Majumder S, Deen MJ (2019). Smartphone sensors for health monitoring and diagnosis. Sensors (Basel).

[4] Chan M, Estève D, Fourniols JY, Escriba C, Campo E (2012). Smart wearable systems: current status and future challenges. Artif Intell Med.

[5] Kim J, Campbell AS, de Ávila BE, Wang J (2019). Wearable biosensors for healthcare monitoring. Nat Biotechnol.

[6] Zhang Y, Hu Y, Jiang N, Yetisen AK (2022). Wearable artificial intelligence biosensor networks. Biosens Bioelectron.

[7] Steinhubl SR, Muse ED, Topol EJ (2013). Can mobile health technologies transform health care?. JAMA.

[8] Cornet VP, Holden RJ (2018). Systematic review of smartphone-based passive sensing for health and wellbeing. J Biomed Inform.

[9] Aranki D, Kurillo G, Yan P, Liebovitz DM, Bajcsy R (2016). Real-time tele-monitoring of patients with chronic heart-failure using a smartphone: lessons learned. IEEE Trans Affect Comput.

[10] Thap T, Chung H, Jeong C, Hwang K-E, Kim H-R, Yoon K-H, Lee J (2016). High-resolution time-frequency spectrum-based lung function test from a smartphone microphone. Sensors (Basel).

[11] Hickey BA, Chalmers T, Newton P, Lin C-T, Sibbritt D, McLachlan CS, Clifton-Bligh R, Morley J, Lal S (2021). Smart devices and wearable technologies to detect and monitor mental health conditions and stress: a systematic review. Sensors (Basel).

[12] Goldberg SB, Lam SU, Simonsson O, Torous J, Sun S (2022). Mobile phone-based interventions for mental health: a systematic meta-review of 14 meta-analyses of randomized controlled trials. PLOS Digit Health.

[13] Palmius N, Tsanas A, Saunders KE, Bilderbeck AC, Geddes JR, Goodwin GM, De Vos M (2017). Detecting bipolar depression from geographic location data. IEEE Trans Biomed Eng.

[14] Faurholt-Jepsen M, Busk J, Þórarinsdóttir H, Frost M, Bardram JE, Vinberg M, Kessing LV (2019). Objective smartphone data as a potential diagnostic marker of bipolar disorder. Aust N Z J Psychiatry.

[15] Burns MN, Begale M, Duffecy J, Gergle D, Karr CJ, Giangrande E, Mohr DC (2011). Harnessing context sensing to develop a mobile intervention for depression. J Med Internet Res.

[16] Arevian AC, Bone D, Malandrakis N, Martinez VR, Wells KB, Miklowitz DJ, Narayanan S (2020). Clinical state tracking in serious mental illness through computational analysis of speech. PLoS One.

[17] Karam ZN, Provost EM, Singh S, Montgomery J, Archer C, Harrington G, Mcinnis MG (2014). Ecologically valid long-term mood monitoring of individuals with bipolar disorder using speech. Proc IEEE Int Conf Acoust Speech Signal Process.

[18] Zhu Y, Jayagopal JK, Mehta RK, Erraguntla M, Nuamah J, McDonald AD, Taylor H, Chang S-H (2020). Classifying major depressive disorder using fNIRS during motor rehabilitation. IEEE Trans Neural Syst Rehabil Eng.

[19] Jacobson NC, Summers B, Wilhelm S (2020). Digital biomarkers of social anxiety severity: digital phenotyping using passive smartphone sensors. J Med Internet Res.

[20] Narziev N, Goh H, Toshnazarov K, Lee SA, Chung K-M, Noh Y (2020). STDD: short-term depression detection with passive sensing. Sensors (Basel).

[21] Mundt JC, Vogel AP, Feltner DE, Lenderking WR (2012). Vocal acoustic biomarkers of depression severity and treatment response. Biol Psychiatry.

[22] Mundt JC, Snyder PJ, Cannizzaro MS, Chappie K, Geralts DS (2007). Voice acoustic measures of depression severity and treatment response collected via interactive voice response (IVR) technology. J Neurolinguistics.

[23] Scott J, Hidalgo-Mazzei D, Strawbridge R, Young A, Resche-Rigon M, Etain B, Andreassen OA, Bauer M, Bennabi D, Blamire AM, Boumezbeur F, Brambilla P, Cattane N, Cattaneo A, Chupin M, Coello K, Cointepas Y, Colom F, Cousins DA, Dubertret C, Duchesnay E, Ferro A, Garcia-Estela A, Goikolea J, Grigis A, Haffen E, Høegh MC, Jakobsen P, Kalman JL, Kessing LV, Klohn-Saghatolislam F, Lagerberg TV, Landén M, Lewitzka U, Lutticke A, Mazer N, Mazzelli M, Mora C, Muller T, Mur-Mila E, Oedegaard KJ, Oltedal L, Pålsson E, Papadopoulos Orfanos D, Papiol S, Perez-Sola V, Reif A, Ritter P, Rossi R, Schulze T, Senner F, Smith FE, Squarcina L, Steen NE, Thelwall PE, Varo C, Vieta E, Vinberg M, Wessa M, Westlye LT, Bellivier F (2019). Prospective cohort study of early biosignatures of response to lithium in bipolar-I-disorders: overview of the H2020-funded R-LiNK initiative. Int J Bipolar Disord.

[24] Ben-Zeev D, Brenner CJ, Begale M, Duffecy J, Mohr DC, Mueser KT (2014). Feasibility, acceptability, and preliminary efficacy of a smartphone intervention for schizophrenia. Schizophr Bull.

[25] Weisel KK, Fuhrmann LM, Berking M, Baumeister H, Cuijpers P, Ebert DD (2019). Standalone smartphone apps for mental health-a systematic review and meta-analysis. NPJ Digit Med.

[26] Taylor S, Jaques N, Nosakhare E, Sano A, Picard R (2020). Personalized multitask learning for predicting tomorrow's mood, stress, and health. IEEE Trans Affect Comput.

[27] Yan S, Hosseinmardi H, Kao H-T, Narayanan S, Lerman K, Ferrara E (2020). Affect estimation with wearable sensors. J Healthc Inform Res.

[28] Firth J, Torous J, Nicholas J, Carney R, Rosenbaum S, Sarris J (2017). Can smartphone mental health interventions reduce symptoms of anxiety? A meta-analysis of randomized controlled trials. J Affect Disord.

[29] Ferreri F, Bourla A, Peretti C-S, Segawa T, Jaafari N, Mouchabac S (2019). How new technologies can improve prediction, assessment, and intervention in obsessive-compulsive disorder (e-OCD): review. JMIR Ment Health.

[30] (2013). Diagnostic And Statistical Manual Of Mental Disorders, Fifth Edition DSM-5.

[31] Calvocoressi L, Libman D, Vegso SJ, McDougle CJ, Price LH (1998). Global functioning of inpatients with obsessive-compulsive disorder, schizophrenia, and major depression. Psychiatr Serv.

[32] Angelakis I, Gooding P, Tarrier N, Panagioti M (2015). Suicidality in obsessive compulsive disorder (OCD): a systematic review and meta-analysis. Clin Psychol Rev.

[33] Hollander E, Doernberg E, Shavitt R, Waterman RJ, Soreni N, Veltman DJ, Sahakian BJ, Fineberg NA (2016). The cost and impact of compulsivity: a research perspective. Eur Neuropsychopharmacol.

[34] Catapano F, Perris F, Masella M, Rossano F, Cigliano M, Magliano L, Maj M (2006). Obsessive-compulsive disorder: a 3-year prospective follow-up study of patients treated with serotonin reuptake inhibitors OCD follow-up study. J Psychiatr Res.

[35] Eisen JL, Sibrava NJ, Boisseau CL, Mancebo MC, Stout RL, Pinto A, Rasmussen SA (2013). Five-year course of obsessive-compulsive disorder. J Clin Psychiatry.

[36] Thorsen AL, Kvale G, Hansen B, van den Heuvel OA (2018). Symptom dimensions in obsessive-compulsive disorder as predictors of neurobiology and treatment response. Curr Treat Options Psychiatry.

[37] Bergfeld IO, Dijkstra E, Graat I, de Koning P, van den Boom BJ, Arbab T, Vulink N, Denys D, Willuhn I, Mocking RJ (2021). Invasive and non-invasive neurostimulation for OCD. Curr Top Behav Neurosci.

[38] Cooper D, Champion SM, Stavropoulos L, Grisham JR (2022). How technology can enhance treatment: a scoping review of clinical interventions for anxiety and obsessive-compulsive spectrum disorders. Br J Clin Psychol.

[39] Munn Z, Peters MD, Stern C, Tufanaru C, McArthur A, Aromataris E (2018). Systematic review or scoping review? Guidance for authors when choosing between a systematic or scoping review approach. BMC Med Res Methodol.

[40] Frank AC, Li R, Peterson B, Narayanan S (2022). Scoping review of wearable and mobile devices used in the assessment and treatment of obsessive-compulsive disorder. OSF Registries.

[41] Peters MD, Godfrey CM, Khalil H, McInerney P, Parker D, Soares CB (2015). Guidance for conducting systematic scoping reviews. Int J Evid Based Healthc.

[42] Aboujaoude E (2017). Three decades of telemedicine in obsessive-compulsive disorder: a review across platforms. J Obsessive Compuls Relat Disord.

[43] van Loenen I, Scholten W, Muntingh A, Smit J, Batelaan N (2022). The effectiveness of virtual reality exposure-based cognitive behavioral therapy for severe anxiety disorders, obsessive-compulsive disorder, and posttraumatic stress disorder: meta-analysis. J Med Internet Res.

[44] Millet B, Touitou Y, Poirier MF, Bourdel MC, Hantouche E, Bogdan A, Olié JP (1998). Plasma melatonin and cortisol in patients with obsessive-compulsive disorder: relationship with axillary temperature, physical activity, and clinical symptoms. Biol Psychiatry.

[45] Alfano CA, Kim KL (2011). Objective sleep patterns and severity of symptoms in pediatric obsessive compulsive disorder: a pilot investigation. J Anxiety Disord.

[46] Drummond LM, Wulff K, Rani RS, White S, Mbanga-Sibanda J, Ghodse H, Fineberg NA (2012). How should we measure delayed sleep phase shift in severe, refractory obsessive-compulsive disorder?. Int J Psychiatry Clin Pract.

[47] Pittig A, Arch JJ, Lam CW, Craske MG (2013). Heart rate and heart rate variability in panic, social anxiety, obsessive-compulsive, and generalized anxiety disorders at baseline and in response to relaxation and hyperventilation. Int J Psychophysiol.

[48] Bussing R, Reid AM, McNamara JP, Meyer JM, Guzick AG, Mason DM, Storch EA, Murphy TK (2015). A pilot study of actigraphy as an objective measure of SSRI activation symptoms: results from a randomized placebo controlled psychopharmacological treatment study. Psychiatry Res.

[49] Donse L, Sack AT, Fitzgerald PB, Arns M (2017). Sleep disturbances in obsessive-compulsive disorder: association with non-response to repetitive transcranial magnetic stimulation (rTMS). J Anxiety Disord.

[50] Jaspers-Fayer F, Lin SY, Belschner L, Mah J, Chan E, Bleakley C, Ellwyn R, Simpson A, McKenney K, Stewart SE (2018). A case-control study of sleep disturbances in pediatric obsessive-compulsive disorder. J Anxiety Disord.

[51] Coles ME, Schubert J, Stewart E, Sharkey KM, Deak M (2020). Sleep duration and timing in obsessive-compulsive disorder (OCD): evidence for circadian phase delay. Sleep Med.

[52] Cox RC, Olatunji BO (2022). Delayed circadian rhythms and insomnia symptoms in obsessive-compulsive disorder. J Affect Disord.

[53] Thierfelder A, Primbs J, Severitt B, Hohnecker CS, Kuhnhausen J, Alt AK, Pascher A, Worz U, Passon H, Seemann J, Ernst C, Lautenbacher H, Holderried M, Kasneci E, Giese MA, Bulling A, Menth M, Barth GM, Ilg W, Hollmann K, Renner TJ (2022). Multimodal sensor-based identification of stress and compulsive actions in children with obsessive-compulsive disorder for telemedical treatment. Annu Int Conf IEEE Eng Med Biol Soc.

[54] Gajadien PT, Postma TS, van Oostrom I, Scheepstra KW, van Dijk H, Sack AT, van den Heuvel OA, Arns M (2023). Sleep predicts the response to rTMS and CBT in patients with OCD: an open label effectiveness study. Int J Clin Health Psychol.

[55] Brown LA, Narine K, Asnanni A, Simon S, Majeed I, Cohen D (2020). Implementation of technology-driven comprehensive physical health assessment in an anxiety specialty clinic: Preliminary pilot study findings. The Behavior Therapist.

[56] Gloster AT, Richard DC, Himle J, Koch E, Anson H, Lokers L, Thornton J (2008). Accuracy of retrospective memory and covariation estimation in patients with obsessive-compulsive disorder. Behav Res Ther.

[57] Tilley PJ, Rees CS (2014). A clinical case study of the use of ecological momentary assessment in obsessive compulsive disorder. Front Psychol.

[58] Rupp C, Falke C, Gühne D, Doebler P, Andor F, Buhlmann U (2019). A study on treatment sensitivity of ecological momentary assessment in obsessive-compulsive disorder. Clin Psychol Psychother.

[59] Way to health. Penn Medicine, Center for Health Care Innovation.

[60] Rupp C, Gühne D, Falke C, Doebler P, Andor F, Buhlmann U (2020). Comparing effects of detached mindfulness and cognitive restructuring in obsessive-compulsive disorder using ecological momentary assessment. Clin Psychol Psychother.

[61] Le Boeuf A (1974). An automated aversion device in the treatment of a compulsive handwashing ritual. J Behav Ther Exp Psychiatry.

[62] Olbrich H, Stengler K, Olbrich S (2016). Smartphone based Geo-Feedback in obsessive compulsive disorder as facilitatory intervention: a case report. J Obs Compuls Relat Disord.

[63] Kashyap H, Reddy P, Mandadi S, Narayanaswamy JC, Sudhir PM, Reddy YC (2019). Cognitive training for neurocognitive and functional impairments in obsessive compulsive disorder: a case report. J Obs Compuls Relat Disord.

[64] Arevian AC, O'Hora J, Rosser J, Mango JD, Miklowitz DJ, Wells KB (2020). Patient and provider cocreation of mobile texting apps to support behavioral health: usability study. JMIR Mhealth Uhealth.

[65] Olsen ST, Basu I, Bilge MT, Kanabar A, Boggess MJ, Rockhill AP, Gosai AK, Hahn E, Peled N, Ennis M, Shiff I, Fairbank-Haynes K, Salvi JD, Cusin C, Deckersbach T, Williams Z, Baker JT, Dougherty DD, Widge AS (2020). Case report of dual-site neurostimulation and chronic recording of cortico-striatal circuitry in a patient with treatment refractory obsessive compulsive disorder. Front Hum Neurosci.

[66] Provenza NR, Sheth SA, Dastin-van Rijn EM, Mathura RK, Ding Y, Vogt GS, Avendano-Ortega M, Ramakrishnan N, Peled N, Gelin LF, Xing D, Jeni LA, Ertugrul IO, Barrios-Anderson A, Matteson E, Wiese AD, Xu J, Viswanathan A, Harrison MT, Bijanki KR, Storch EA, Cohn JF, Goodman WK, Borton DA (2021). Long-term ecological assessment of intracranial electrophysiology synchronized to behavioral markers in obsessive-compulsive disorder. Nat Med.

[67] Hawley LL, Rector NA, DaSilva A, Laposa JM, Richter MA (2021). Technology supported mindfulness for obsessive compulsive disorder: self-reported mindfulness and EEG correlates of mind wandering. Behav Res Ther.

[68] Hawley LL, Rector NA, Richter MA (2021). Technology supported mindfulness for obsessive compulsive disorder: the role of obsessive beliefs. J Anxiety Disord.

[69] Fridgeirsson EA, Bais MN, Eijsker N, Thomas RM, Smit DJ, Bergfeld IO, Schuurman PR, van den Munckhof P, de Koning P, Vulink N, Figee M, Mazaheri A, van Wingen GA, Denys D (2023). Patient specific intracranial neural signatures of obsessions and compulsions in the ventral striatum. J Neural Eng.

[70] Motti VG, Caine K, Brenner M, Christin N, Johnson B, Rohloff K (2015). Users’ privacy concerns about wearables. Financial Cryptography and Data Security.

[71] Bauer M, Glenn T, Geddes J, Gitlin M, Grof P, Kessing LV, Monteith S, Faurholt-Jepsen M, Severus E, Whybrow PC (2020). Smartphones in mental health: a critical review of background issues, current status and future concerns. Int J Bipolar Disord.

[72] Torous J, Wisniewski H, Liu G, Keshavan M (2018). Mental health mobile phone app usage, concerns, and benefits among psychiatric outpatients: comparative survey study. JMIR Ment Health.

[73] Hathaliya JJ, Tanwar S (2020). An exhaustive survey on security and privacy issues in Healthcare 4.0. Comput Commun.

[74] Narayanan S, Georgiou PG (2013). Behavioral signal processing: deriving human behavioral informatics from speech and language: computational techniques are presented to analyze and model expressed and perceived human behavior-variedly characterized as typical, atypical, distressed, and disordered-from speech and language cues and their applications in health, commerce, education, and beyond. Proc IEEE Inst Electr Electron Eng.

[75] Onnela J-P, Rauch SL (2016). Harnessing smartphone-based digital phenotyping to enhance behavioral and mental health. Neuropsychopharmacology.

[76] Torous J, Onnela J-P, Keshavan M (2017). New dimensions and new tools to realize the potential of RDoC: digital phenotyping via smartphones and connected devices. Transl Psychiatry.

[77] Adler DA, Ben-Zeev D, Tseng VW, Kane JM, Brian R, Campbell AT, Hauser M, Scherer EA, Choudhury T (2020). Predicting early warning signs of psychotic relapse from passive sensing data: an approach using encoder-decoder neural networks. JMIR Mhealth Uhealth.

[78] Saeb S, Zhang M, Karr CJ, Schueller SM, Corden ME, Kording KP, Mohr DC (2015). Mobile phone sensor correlates of depressive symptom severity in daily-life behavior: an exploratory study. J Med Internet Res.

[79] Kathan A, Harrer M, Küster L, Triantafyllopoulos A, He X, Milling M, Gerczuk M, Yan T, Rajamani ST, Heber E, Grossmann I, Ebert DD, Schuller BW (2022). Personalised depression forecasting using mobile sensor data and ecological momentary assessment. Front Digit Health.

[80] Jacobson NC, Feng B (2022). Digital phenotyping of generalized anxiety disorder: using artificial intelligence to accurately predict symptom severity using wearable sensors in daily life. Transl Psychiatry.

[81] Demiris G, Corey Magan KL, Parker Oliver D, Washington KT, Chadwick C, Voigt JD, Brotherton S, Naylor MD (2020). Spoken words as biomarkers: using machine learning to gain insight into communication as a predictor of anxiety. J Am Med Inform Assoc.

[82] McKernan EP, Kumar M, Di Martino A, Shulman L, Kolevzon A, Lord C, Narayanan S, Kim SH (2022). Intra-topic latency as an automated behavioral marker of treatment response in autism spectrum disorder. Sci Rep.

[83] Lahiri R, Nasir M, Kumar M, Kim SH, Bishop S, Lord C, Narayanan S (2022). Interpersonal synchrony across vocal and lexical modalities in interactions involving children with autism spectrum disorder. JASA Express Lett.

[84] Barron DS, Baker JT, Budde KS, Bzdok D, Eickhoff SB, Friston KJ, Fox PT, Geha P, Heisig S, Holmes A, Onnela J, Powers A, Silbersweig D, Krystal JH (2021). Decision models and technology can help psychiatry develop biomarkers. Front Psychiatry.

[85] Schumacher S, Kent N (2020). 8 charts on internet use around the world as countries grapple with COVID-19. Pew Research Center.

[86] Wike R, Silver L, Fetterolf J, Huang C, Austin S, Clancy L, Gubbala S (2022). Internet, smartphone and social media use. Pew Research Center.

[87] Naveed S, Waqas A, Chaudhary AM, Kumar S, Abbas N, Amin R, Jamil N, Saleem S (2020). Prevalence of common mental disorders in South Asia: a systematic review and meta-regression analysis. Front Psychiatry.

[88] Cai H, Jin Y, Liu R, Zhang Q, Su Z, Ungvari GS, Tang YL, Ng CH, Li XH, Xiang YT (2023). Global prevalence of depression in older adults: a systematic review and meta-analysis of epidemiological surveys. Asian J Psychiatr.

[89] Williams M, Powers M, Yun YG, Foa E (2010). Minority participation in randomized controlled trials for obsessive-compulsive disorder. J Anxiety Disord.

[90] Wetterneck CT, Little TE, Rinehart KL, Cervantes ME, Hyde E, Williams M (2012). Latinos with obsessive-compulsive disorder: mental healthcare utilization and inclusion in clinical trials. J Obsessive Compuls Relat Disord.

[91] Wong CK, Ho DT, Tam AR, Zhou M, Lau YM, Tang MO, Tong RC, Rajput KS, Chen G, Chan SC, Siu CW, Hung IF (2020). Artificial intelligence mobile health platform for early detection of COVID-19 in quarantine subjects using a wearable biosensor: protocol for a randomised controlled trial. BMJ Open.

[92] Maaß L, Freye M, Pan CC, Dassow HH, Niess J, Jahnel T (2022). The definitions of health apps and medical apps from the perspective of public health and law: qualitative analysis of an interdisciplinary literature overview. JMIR Mhealth Uhealth.

[93] Simon DA, Shachar C, Cohen IG (2022). Unsettled liability issues for "prediagnostic" wearables and health-related products. JAMA.

